# The mechanism and application of computer‐assisted full facial skin imaging systems

**DOI:** 10.1002/ski2.320

**Published:** 2023-12-04

**Authors:** Yue Zhang, Ruoxin Pan, Duoduo Gu, Xiaoqi Meng, Tingwei Liu, Yang Xu

**Affiliations:** ^1^ Department of Dermatology The First Affiliated Hospital of Nanjing Medical University Nanjing China

## Abstract

Computer‐assisted full facial imaging systems are currently among the most widely used skin analysis instruments in dermatology and medical cosmetology. These systems offer objective quantitative evaluation of facial skin conditions, and as they are non‐invasive, play an important role in assessing dermatological conditions such as pigmentation, inflammation, vascular diseases, skin texture, the severity of ageing, and therapeutic follow‐up. Although computer‐assisted full facial imaging systems enable quantitative analysis in the scope of medical treatment and cosmetic evaluation, their results may considerably vary because of the influence of environmental and postural factors for improper operation. Furthermore, manual observation is sometimes necessary for experimental work for more accuracy, and familiarity with the imaging principles and application points is necessary to best apply this technique. This report aims to discuss and interpret these systems' imaging mechanisms and explore the primary issues with their application.

## Graphical abstract

Computer‐assisted full facial skin imaging systems have been widely used in both clinical and research application to offer objective quantitative evaluation of surface and subsurface facial skin conditions. These systems are non‐invasive, play an important role in assessing dermatological conditions such as pigmentation, inflammation, vascular diseases, skin texture, the severity of ageing, and therapeutic follow‐up. However, non‐standard operation or inaccurate interpretation of the data may lead to deviations and inconsistencies of the final results. This review aims to discuss and interpret these systems' imaging mechanisms and explore the primary issues with their application.

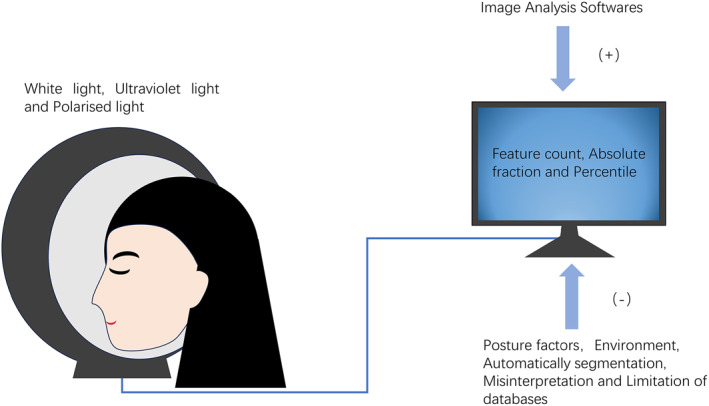

**What is already known about this topic?**

- Computer‐assisted full facial skin imaging systems have begun to be widely used in both clinical and research application to record and measure surface and subsurface of facial skin in recent years.

**What does this study add?**

- This purpose of this study is to elaborate on the main points of computer‐assisted full facial skin imaging systems to optimise its application.

## 1 INTRODUCTION

Computer‐assisted full facial skin imaging systems are currently among the most widely used facial skin analysis instruments in dermatology and medical cosmetology. These systems are sometimes called skin image analysers, intelligent skin detectors, or multi‐spectral facial image analysers. Compared with non‐invasive point measurement optical devices, computer‐assisted full facial skin imaging systems focus on the overall facial skin characteristics.^1^, ^2^ Though these systems can provide reproducible and reliable digital images and quantitative data for facial skin lesion evaluation, the results may vary considerably for improper operation such as inconsistency of environmental lighting and view angles during follow‐ups.^3^

The common commercial full facial skin imaging systems include VISIA^®^ (Canfeld Scientific Inc.),^4^ OBSERV^®^ (Sylton Inc.),^5^ CSKIN^®^ (Yanyun Technology Co., Ltd.,),^6^ IPP^®^ (Media Cybernetics Inc.),^7^ and so on, which may share the similar basic imaging apparatus and the underlying mechanism including light sources, image modes, and data provided by the system.

## 2 LIGHT SOURCES

There are three main light sources used in these systems: white light, ultraviolet (UV) light, and polarised light.

### 2.1 Red, green, and blue light

Red, green, and blue (RGB) light is simulated by artificial light and can be perceived by the naked eye. The images collected comprised three primary colours: red, blue, and green. The RGB image is a three‐dimensional collection of data in which each pixel contains information on each colour channel. The light source can be either a xenon flash or a light‐emitting diode.

### 2.2 Ultraviolet light and/or Wood's light

**FIGURE 1:**
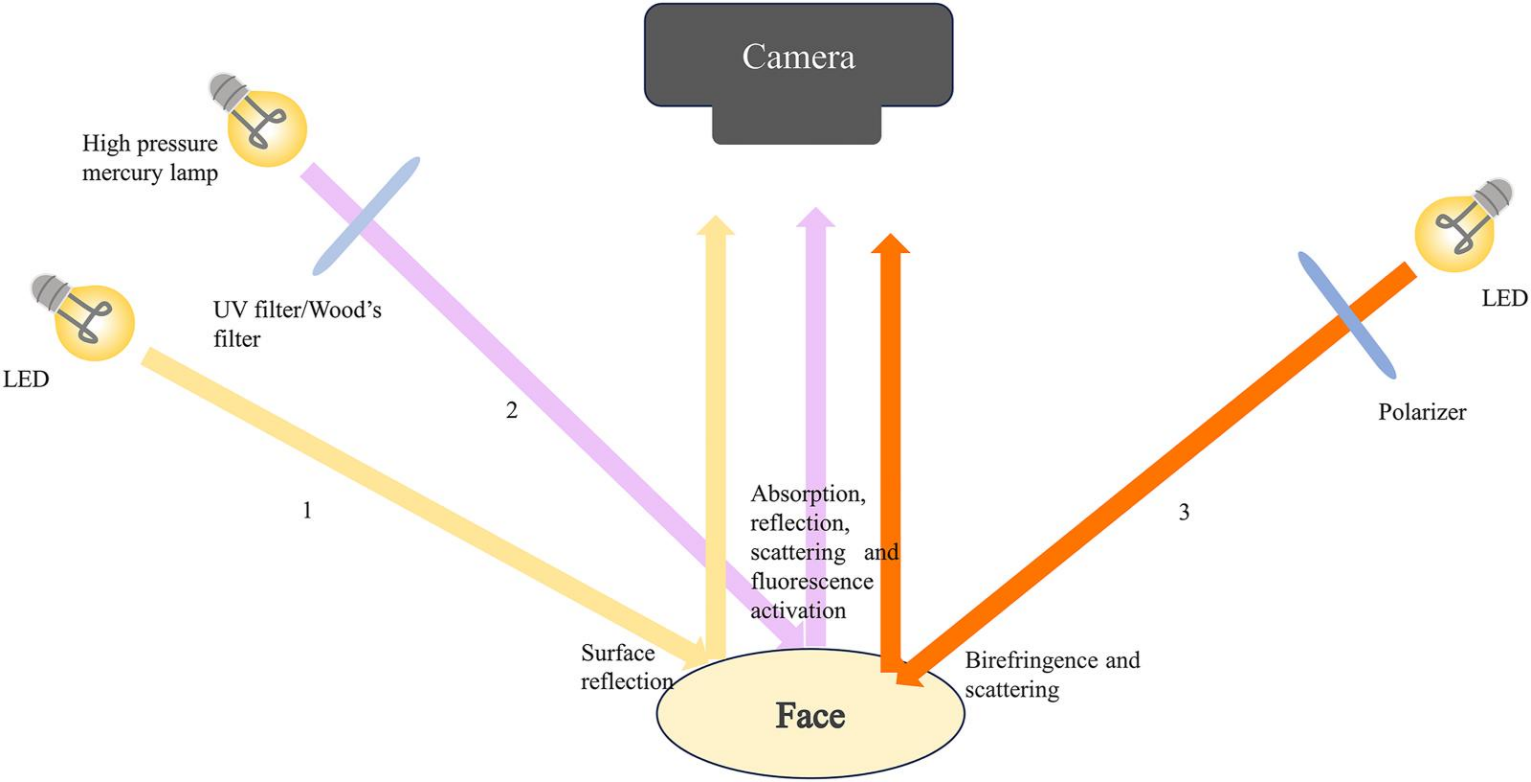
Three main light sources: the white light (1, yellow arrow), ultraviolet A and/or Wood's light (2, purple allow) and polarised light (3, orange arrow).

The UV light in imaging systems is primarily ultraviolet A (UVA) with a wavelength between 320 and 400 nm, also known as black light (Figure 1).^8^ The spectrum of Wood's light is also between 320 and 400 nm with a peak at 365 nm, which is within the spectrum of UVA and shares the same underlying mechanism of skin imaging.

The basic principles of UV light imaging include absorption, reflection, scattering, and fluorescence activation of different chromophores in the skin, including melanin, elastin, collagen (pyridinoline crosslinks), aromatic amino acids (predominantly tryptophan and its oxidative products), nicotinamide adenine dinucleotide, and others.^9^

Melanin absorbs UV light without excitation by UV‐induced fluorescence (UVF), resulting in the hyperpigmented skin area appearing darker than the surrounding area under UV light, which is then presented in grayscale processed by computer. *Propionibacterium acne* emits a fluorescence peaking at approximately 635 nm, while *Staphylococcus epidermidis*, cultured anaerobically and then exposed to air, emits fluorescence peaking at 620 nm; both may be manifested as red UVF in porphyrin images. No red UVF was reported to be directly emitted from sebum or sebaceous glands.^10^ With an excitation wavelength of 355 nm, the fluorescence spectrum of pityrialactone (a metabolism of *Malassezia furfur*) in acetonitrile is blue with a peak at 480 nm and is brighter in intensity,^11^ which likely explains the blue‐white fluorescence UVF characterised the colonisation of *Malassezia.* ^12^

### 2.3 Polarised light

Polarised light commonly used in skin imaging systems includes parallel‐polarised and cross‐polarised light with a wavelength within the visible light range. While parallelpolarized light is often applied to highlight the details of the skin surface, including fine lines, pores, and spots, cross‐polarised light emphasises subepidermal characteristics including pigmentation and blood vessels (Figure 1).^13^

## 3 IMAGE MODES

Though there are differences between the image systems regarding the number of presented image modes (8–12 modes), they are all essentially superimposed, calculated, and processed based on the images taken under the aforementioned light sources. The basic common image modes include white light, UV spot, brown spot, red area, and UVF mode. Other image modes may also be provided to highlight specific features after further data calculation, ultimately enhancing the intuitiveness.

The patented algorithms in some imaging systems enable a combination of images taken with white light and images taken with polarised light to produce images in red and brown modes, with the brown spot mode presenting mainly hyperpigmentation and red area pattern representing telangiectasia, inflammation, and vascular lesions. In most cases, the two modes are used for different purposes to differentiate between pigmentation and vascular lesions.

While *Malassezia* primarily emits blue‐white fluorescence, and *Propionibacterium acne* together with *S. epidermidis* can be detected by the red fluorescence in the UVF mode. Both are enriched in sebum‐rich areas; thus, this pattern can be used to monitor the function of the pilosebaceous unit indirectly.

## 4 DATA MEASUREMENT

After taking pictures, the system automatically encircles (or the operator manually circles) the area to be analysed according to the corresponding facial region, providing three sets of data with corresponding features: feature count, absolute fraction, and percentile. Feature count is the number of evaluated features, regardless of size or intensity, which indicates changes in the number of features. Absolute scores provide an overall feature assessment, including number, size, area, and intensity. Percentile is calculated by comparing the subjects' characteristics against a database of people of the same sex, age, and skin type, providing a baseline assessment of individual skin characteristics measured in this population, which is related to the system's database.

## 5 CLINICAL APPLICATION

Based on clinical images and data provided by computer‐assisted facial imaging systems, the severity and treatment efficacy of various facial skin diseases can be assessed. A combination of white light images with UV spot images or brown spot mode is commonly applied for severity evaluation and follow‐up of pigmented facial skin diseases^14^ such as dull skin,^15^, ^16^ freckles,^17^ melasma,^18^, ^19^ post‐inflammatory pigmentation,^20^, ^21^ and Riehl melanosis.^22^ In the study of facial inflammatory dermatoses, white light mode and red area mode with or without UV light images are frequently combined to evaluate the condition, including acne,^23^, ^24^ rosacea,^4^, ^6^, ^25^, ^26^, ^27^ rosacea‐like dermatitis induced by steroids,^28^ and seborrhoeic dermatitis^29^ in addition to facial vascular anomalies such as port wine stains.^30^, ^31^ Evaluation of the effects of anti‐ageing treatments and depression scar treatment mostly involves assessing facial skin texture, wrinkles, pores, and skin texture, usually using a white light mode.^32^, ^33^, ^34^, ^35^, ^36^, ^37^, ^38^ The systems have also been used to assess the treatment efficiency of striae distensae,^39^ the cleanliness of facial makeup products^40^ sunscreen application,^41^, ^42^ and the effect of face masks on makeup.^43^

## 6 KEY NOTES DURING THE PROCESS OF APPLICATION

Even though the computer‐assisted full facial imaging systems provide a non‐invasive objective quantitative evaluation of skin conditions, some disadvantages remain that may interfere with the scientific accurate application and interpretation.

**FIGURE 2:**
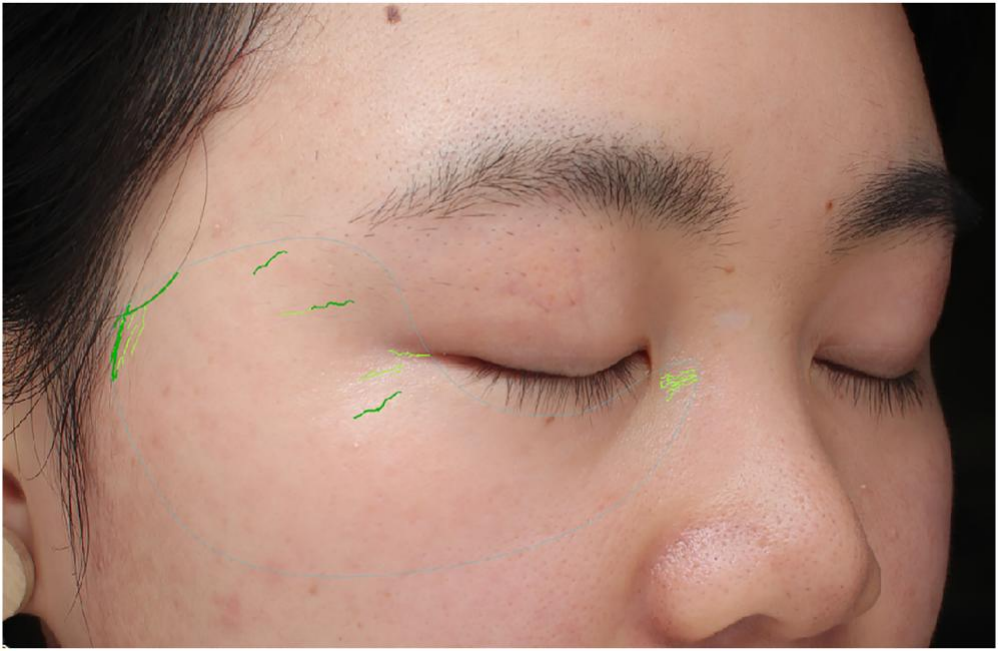
In the white light mode, unruly hair may affect the automatic selection of the areas processed as wrinkles (green lines).

Wrinkles and skin texture are presented and analysed in white light mode. The long, thin, slender folds and creases are analysed as wrinkles under white light. However, untidy hair and expression during shooting may influence the results (Figure 2), which should be cautioned by dermatologists in the process of data analysis.

**FIGURE 3:**
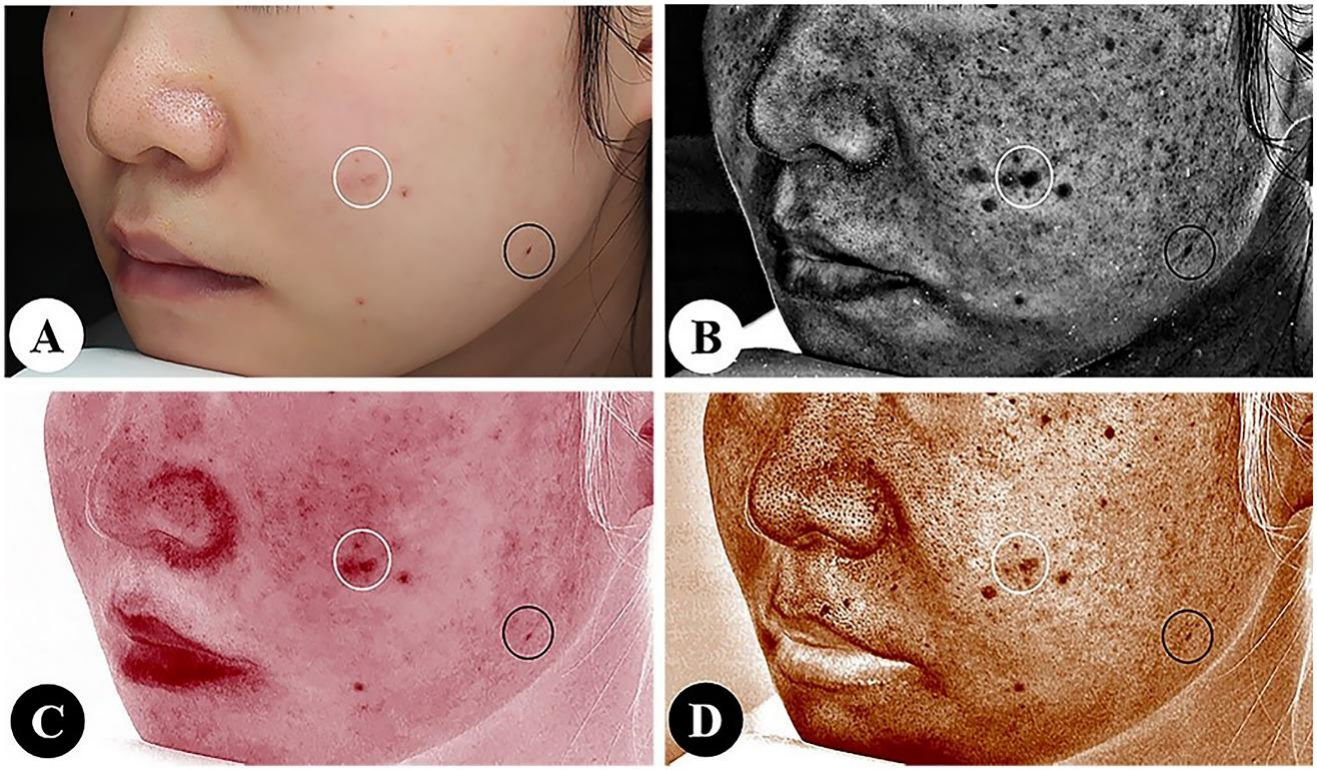
It is necessary to combine the images in white light mode (a) to differentiate facial acne (white circles) from nevus (black circles) in ultraviolet spot (b), red area (c) and brown spot (d) modes.

In most cases, the brown spot and the red area modes can differentiate pigmentation from blood vessels. However, there may be some overlap between the two because of the interference of the optical signal itself. As a result, the manual combination of images taken by white light is still required for comprehensive analysis (Figure 3).

**FIGURE 4:**
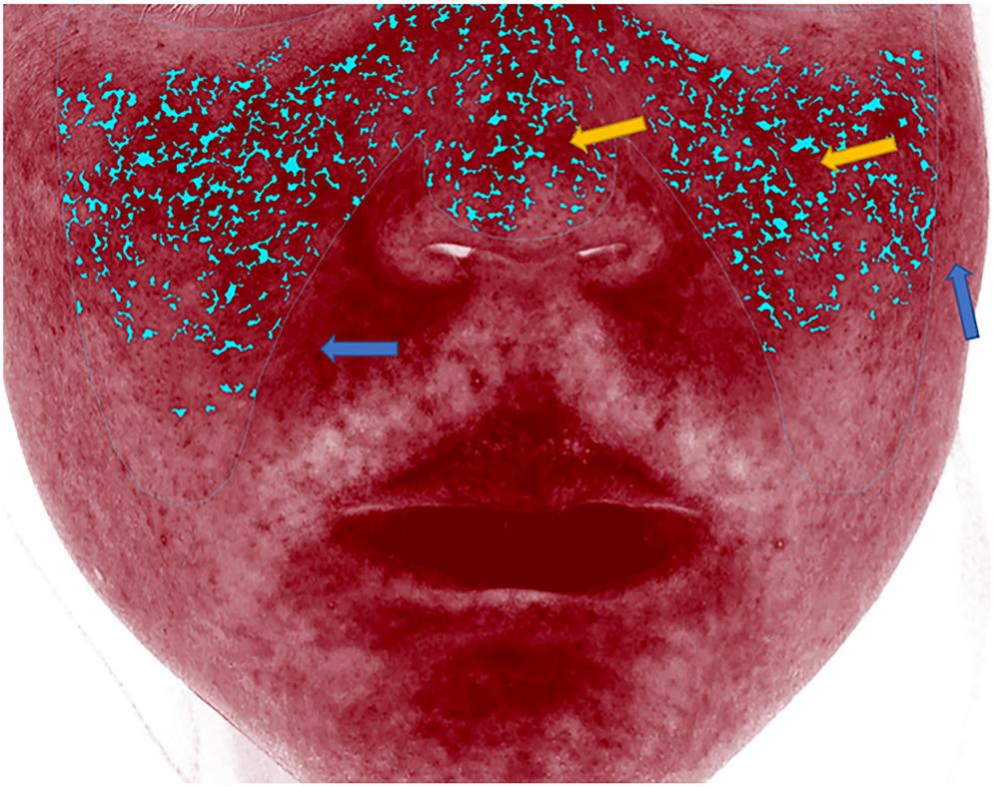
Erythema segmented automatically by the VISIA^®^ system (Blue dotted area), with diffuse erythema not thoroughly segmented (yellow arrows) and erythema not automatically fully recognised (blue arrows).

Erythema cannot be automatically segmented or recognised fully in most of these systems, especially when presented in a diffuse or gradient manner, making the data imprecise (Figure 4). If accurate or specific data is required during scientific research and clinical evaluation, the photos should be exported and analysed using particular software systems such as ImageJ,^5^ and a new algorithm may be needed.^44^, ^45^

**FIGURE 5:**
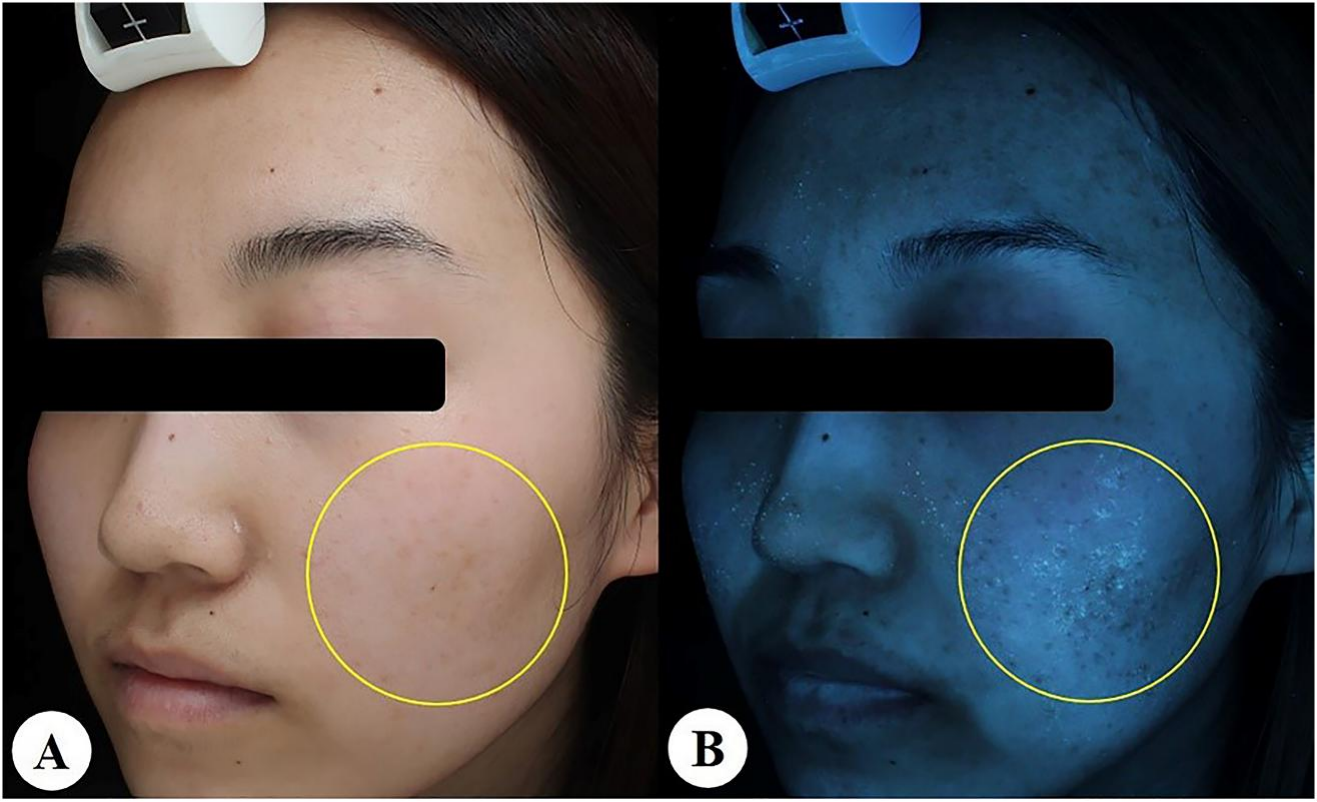
Facial exudate scabs shown in white light mode (a) can be fluorescently blue‐green (yellow circle) in ultraviolet‐induced fluorescence mode (b).

In addition, some specific substances on the skin surface can be observed clearly in UVF mode, including serous scabs, paper towels, certain drugs, cosmetics, and residual fluorescent agents of sunscreen or other substances (Figure 5), which should be considered during the process of analysing the UVF images.

All advantages and disadvantages of these systems are listed in Table 1, with keynotes to be cautioned by practitioners.

**TABLE 1:** Characteristics of the images provided by computer‐assisted full facial skin imaging systems.

	Highlights	Notes
White light images	Wrinkle and skin texture	Unruly hair may affect the automatic selection of the areas processed as wrinkles
UV spot images	Pigmentation on skin surface	Not equal to the epidermal hyperpigmentation
Browns spot images	Subsurface pigmentation	Not equal to the dermal pigmentation; May also include information from haemoglobin
Red area images	Blood vessels and haemoglobin in the dermal papilla	May also contain information of subsurface pigmentation
UVF images	Colonisation of *Propionibacterium acnes Staphylococcus epidermidis* and *Malassezia*	Not refer to sebum directly
Data	Three kinds of data: feature count, absolute fraction, and percentile	The limitation of the automatic segmentation and the database used

Abbreviation: UVF, UV‐induced fluorescence.

## 7 CONCLUSION

In recent years, computer‐assisted full facial imaging systems have been widely used to evaluate pigmentation, inflammation, vascular diseases, skin texture, severity of ageing, and therapeutic follow‐up for higher accuracy and more objective and precise data. Compared with non‐invasive point measurement optical devices, computer‐assisted full facial imaging systems allow reproducible image acquisition and both qualitative and quantitative images of widely distributed skin lesions. Familiarity with the imaging principles and application points is helpful for better utilisation of this technique. With the development of technology, algorithms, and the enrichment of databases, computer‐assisted full facial imaging systems will be more useful to improve the clinical evaluation efficiency of facial skin lesions in dermatology and medical cosmetology in the future.

## CONFLICT OF INTEREST STATEMENT

The authors declare no conflicts of interest.

## AUTHOR CONTRIBUTIONS

**Yue Zhang**: Conceptualization (equal); data curation (equal); formal analysis (equal); investigation (equal); methodology (equal); resources (equal); software (equal); validation (equal); visualization (equal); writing—original draft (lead); writing—review and editing (equal). **Ruoxin Pan**: Data curation (equal); formal analysis (supporting); project administration (equal); resources (equal); software (equal). **Duoduo Gu**: Data curation (equal); formal analysis (supporting); investigation (supporting); resources (equal); software (equal); supervision (equal). **Xiaoqi Meng**: Data curation (supporting); formal analysis (supporting); resources (supporting); software (supporting). **Tingwei Liu**: Data curation (equal); formal analysis (equal); resources (supporting); software (supporting). **Yang Xu**: Conceptualization (equal); data curation (equal); formal analysis (equal); investigation (equal); methodology (equal); supervision (equal); validation (equal); writing—original draft (equal); writing—review and editing (equal).

## ETHICS STATEMENT

Not applicable.

## Data Availability

The data underlying this article will be shared on reasonable request to the corresponding author.
